# Pulmonary Arteriovenous Pressure Gradient and Time-Averaged Mean Velocity of Small Pulmonary Arteries Can Serve as Sensitive Biomarkers in the Diagnosis of Pulmonary Arterial Hypertension: A Preclinical Study by 4D-Flow MRI

**DOI:** 10.3390/diagnostics12010058

**Published:** 2021-12-28

**Authors:** Ali Nahardani, Simon Leistikow, Katja Grün, Martin Krämer, Karl-Heinz Herrmann, Andrea Schrepper, Christian Jung, Sara Moradi, Paul Christian Schulze, Lars Linsen, Jürgen R. Reichenbach, Verena Hoerr, Marcus Franz

**Affiliations:** 1 Medical Physics Group, Institute of Diagnostic and Interventional Radiology, Jena University Hospital, Friedrich Schiller University Jena, 07747 Jena, Germany; nahardani.ali@gmail.com (A.N.); Martin.Kraemer@med.uni-jena.de (M.K.); Karl-Heinz.Herrmann@med.uni-jena.de (K.-H.H.); Juergen.Reichenbach@med.uni-jena.de (J.R.R.); 2 Heart Center Bonn, Department of Internal Medicine II, University Hospital Bonn, 53127 Bonn, Germany; sara.moradi.567@gmail.com; 3 Department of Mathematics and Computer Science, Institute of Computer Science, Westfälische Wilhelms-Universität Münster, 48149 Munster, Germany; simon.leistikow@uni-muenster.de (S.L.); linsen@uni-muenster.de (L.L.); 4 Department of Internal Medicine I, Division of Cardiology, Angiology, Pneumology, and Intensive Medical Care, Jena University Hospital, 07747 Jena, Germany; Katja.Gruen@med.uni-jena.de (K.G.); Christian.Schulze@med.uni-jena.de (P.C.S.); Marcus.Franz@med.uni-jena.de (M.F.); 5 Department of Cardiothoracic Surgery, Jena University Hospital, 07747 Jena, Germany; Andrea.Schrepper@med.uni-jena.de; 6 Department of Internal Medicine, Division of Cardiology, University Hospital Düsseldorf, 40225 Dusseldorf, Germany; Christian.Jung@med.uni-duesseldorf.de; 7 Translational Research Imaging Center (TRIC), Clinic for Radiology, University Hospital Münster, 48149 Munster, Germany

**Keywords:** pulmonary hypertension, cardiac magnetic resonance, treatment response, hemodynamics, 4D-flow

## Abstract

(1) Background: Pulmonary arterial hypertension (PAH) is a serious condition that is associated with many cardiopulmonary diseases. Invasive right heart catheterization (RHC) is currently the only method for the definitive diagnosis and follow-up of PAH. In this study, we sought a non-invasive hemodynamic biomarker for the diagnosis of PAH. (2) Methods: We applied prospectively respiratory and cardiac gated 4D-flow MRI at a 9.4T preclinical scanner on three different groups of Sprague Dawley rats: baseline (*n* = 11), moderate PAH (*n* = 8), and severe PAH (*n* = 8). The pressure gradients as well as the velocity values were analyzed from 4D-flow data and correlated with lung histology. (3) Results: The pressure gradient between the pulmonary artery and vein on the unilateral side as well as the time-averaged mean velocity values of the small pulmonary arteries were capable of distinguishing not only between baseline and severe PAH, but also between the moderate and severe stages of the disease. (4) Conclusions: The current preclinical study suggests the pulmonary arteriovenous pressure gradient and the time-averaged mean velocity as potential biomarkers to diagnose PAH.

## 1. Introduction

Pulmonary hypertension (PH) is a life-threatening condition that is defined as an increase in mean pulmonary artery pressure above 20 mmHg when measured by right heart catheterization (RHC) at rest [1,2]. Clinically, pulmonary arterial hypertension (PAH) is categorized as the first group in the PH classification, which is described in detail in the clinical guidelines of the European Society of Cardiology [1]. The clinical manifestations of PAH are often nonspecific and usually arise from right ventricular dysfunction in advanced stages [1,3]. Routine blood gas and respiratory function tests, electrocardiography (ECG), chest X-ray, as well as blood and serological tests can produce different results for a variety of conditions and indicate a number of possible underlying diseases [1,4]. Therefore, further evaluations by means of additional diagnostic tools are recommended for differential diagnosis. RHC is the diagnostic standard for the definite diagnosis of PH, the assessment of the severity of hemodynamic impairment in its course and for further follow-ups [1,5,6,7]. However, there are a variety of pitfalls when assessing and interpreting the parameters determined by RHC; for example, the measurement of the pulmonary artery wedge pressure at an over- or under-wedge position can lead to incorrect results [8]. In addition, as an invasive diagnostic tool, RHC can cause intra- or post-procedure complications, such as hematomas, pulmonary artery ruptures, or damage to the electrical conduction system of the heart [9]. Furthermore, it is contraindicated under certain circumstances such as in mechanical tricuspid or pulmonary valve replacement, right heart masses, etc. [7]. In contrast to RHC, transthoracic echocardiography (TTE) is a non-invasive method that cannot directly measure pulmonary vascular resistance or any other related indices to vascular pressure, but plays an important role in assessing the likelihood of PAH, the development of right heart failure, or the necessity for catheterization in symptomatic patients [1,10]. Nuclear imaging and computed tomography techniques are also indicated in pulmonary hypertension assessments [11,12,13], but suffer from high effective dose exposures. Cardiac magnetic resonance (CMR) can improve patient care in clinics substantially [14] as it is the gold standard for quantifying right-heart function [15,16] and is useful to PH evaluations [5,15,17]. In recent years, the development of 4D-flow CMR has opened a new direction for the qualitative and quantitative assessment of cardiovascular hemodynamics and function [18,19,20]. In previous studies, various CMR hemodynamic indices (such as peak velocity, blood flow, etc.) were compared to RHC [19,21,22]. However, none of the evaluated biomarkers correlated very strongly with the catheterization results. Among all the parameters, the most promising non-invasive hemodynamic index was the time-averaged mean velocity of the main pulmonary artery [19]. For this purpose, we evaluated this index in the small pulmonary arteries in 4D-flow and its correlation with histology to assess its suitability for diagnosing PAH. In addition, we calculated the pulmonary arteriovenous pressure gradient (i.e., the pressure gradient between the pulmonary small arteries and veins of the unilateral side) in all the experimental groups to evaluate its diagnostic utility for PAH. To confirm the influence of the experimental PAH model on heart mechanics, we calculated tricuspid annular plane systolic excursion (TAPSE) by means of TTE and right-ventricular ejection fraction (RVEF) by using MRI. Finally, a comprehensive correlation analysis of all the CMR findings in relation to the histology results was performed.

## 2. Materials and Methods

**Figure 1:**
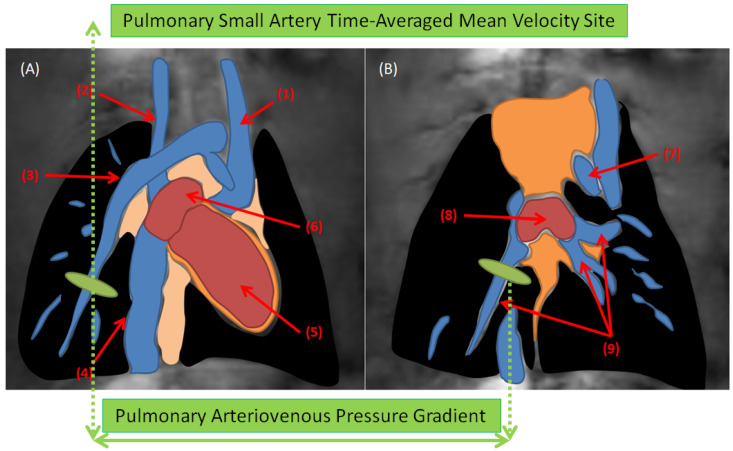
A schematic representation showing the region of interests (ROI) for the calculation of pulmonary arteriovenous pressure gradients and pulmonary small arteries’ time-averaged mean velocities. The green label in (**A**) shows ROI on a small pulmonary artery, and in (**B**) depicts ROI on the ipsilateral pulmonary vein (Annotations: (1) left superior vena cava (2) right superior vena cava (3) right pulmonary artery along its small branch (4) inferior vena cava (5) right ventricle (6) right atrium (7) aorta (8) left atrium (9) pulmonary veins).

- **Animal model**: A total of 27 male Sprague Dawley rats (Charles River Laboratories, Sulzfeld, Germany) with an average weight of 330 ± 39.9 g were included in the study and divided into three different experimental groups: (A) Baseline (*n* = 11, with a nuchal subcutaneous injection of 300 µL NaCl 0.9%); (B) Severe PAH (*n* = 8, with a single-dose subcutaneous injection of 60 mg/kg monocrotaline—Carl Roth, Karlsruhe, Germany—dissolved in 300 µL NaCl 0.9%); and (C) Moderate PAH (*n* = 8, with the same medication as the severe PAH group and an additional oral application of 15 mg/kg macitentan—Actelion Pharmaceuticals Ltd., Allschwil, Switzerland—from day 14 to 28). All invasive and non-invasive diagnostic procedures were performed 4 weeks after disease induction. The general anesthesia protocol was chosen depending on the diagnostic procedure: all the non-invasive procedures were conducted under inhalation of isoflurane ~3% (due to the long tubing between the vaporizers and the MRI system) to keep the animals’ respiration in the range of 30 to 50 per minute. Perfusion was carried out under deep anesthesia with the intra-peritoneal administration of a single dose of 100 mg/kg body weight of ketamine (volume 450 µL) and 10 mg/kg body weight of xylazine (volume 250 µL). All the experiments were conducted in accordance with approved ethical guidelines (see the section on the Institutional Review Board Statement).
- **Cardiac Magnetic Resonance:** Prospectively, a cardiac and respiratory triggered 4D-flow stack-of-stars phase-contrast sequence was performed on a 9.4 T BioSpec USR 94/20 imaging scanner with ParaVision 6.0.1 software (Bruker, Ettlingen, Germany). All the animals were examined by a vendor-supplied 72 mm-diameter quadrature volume resonator with the following sequence parameters. Flow encoding scheme: HADAMARD; TR = 10 ms; TE = 1.1 ms; FA = 10°; BW = 100 kHz; under-sampling factor = 1.4–1.6; averages = 1; resolution = (375 × 375 × 375) µm^3^; VENC = 75–200 cm/s. The data were reconstructed offline using regridding with iterative sampling density estimation. All the functional and hemodynamic parameters were quantified using manual region-of-interests (ROI). The time-averaged mean-velocities were calculated in the large (main, right and left pulmonary arteries) and small pulmonary arteries. Furthermore, the pulmonary arteriovenous pressure gradients were calculated through the use of modified Bernoulli’s equation from 4D-flow data (Figure 1). All the parameters investigated by MRI are listed in Table 1 and Table 2.
- **Transthoracic echocardiography:** TTE was performed in all animals before CMR with a high-resolution ultrasound imaging system Vevo-770 (Visual Sonics, Toronto, ON, Canada) using a rodent specific 17 MHz probe. The recorded TTE movies were analyzed by an experienced cardiologist and the calculated parameter is listed in Table 2.
- **Histological assessment of lungs:** The formalin-fixed, paraffin-embedded, and H&E-stained lung tissue sections were evaluated by two experienced scientists. PAH-associated tissue damage of the lungs was evaluated semi-quantitatively using an established and validated sum-score system of our group [23,24]. The list of the evaluated histological indices is summarized in Table 3. According to the scoring system, atelectasis area (AA), emphysema area (EA), peribronchial artery media hypertrophy (PAMH), peribronchial artery perivascular cellular edema (PAPCE), and small artery media hypertrophy (SAMH) were evaluated and scored individually. Subsequently, all the scores were added up and summarized by an index called “Lung assessment sum-score”, or LASS, reflecting the overall pulmonary tissue changes due to PAH.
- **Statistical Analysis:** The pairwise Mann–Whitney U test was performed to compare the means of various variables among the experimental groups statistically with a *p*-value significance threshold of 5%. In addition, the Spearman correlation (between the scalar and ordinal variables) and Pearson correlation (between two scalar variables) tests were used for correlation analysis between two different parameters (Table 4). The strength of the correlation coefficients was classified according to the following scale: very strong correlation (r = 0.90 to 1.0), strong correlation (r = 0.70 to 0.90), moderate correlation (r = 0.50 to 0.70), low and negligible correlation (r < 0.50) [25].

## 3. Results

**Figure 2:**
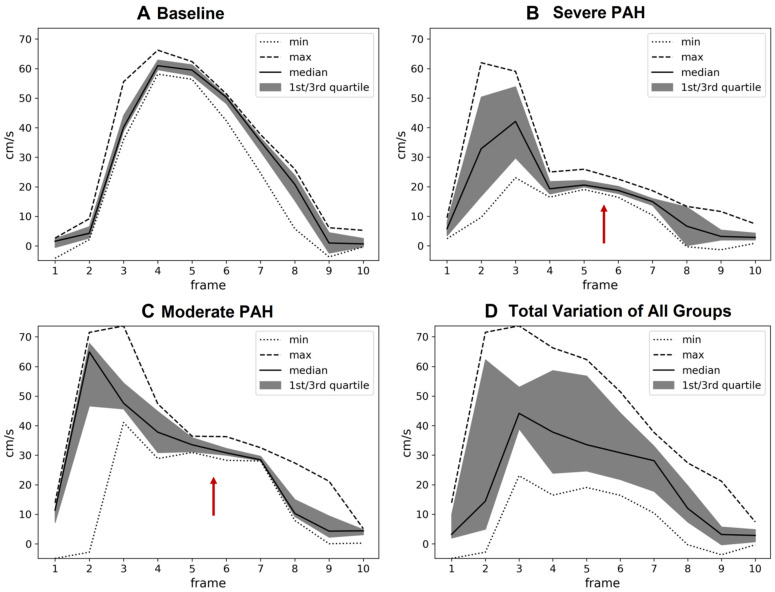
(**A**–**C**) Intra- and (**D**) inter-experimental group variability assessment of the velocity-time curve through a cross-section drawn in the distal portion of the main pulmonary artery, i.e., proximal to the pulmonary bifurcation. Notches are indicated by red arrows. Each cardiac frame corresponds to a temporal resolution of 10 ms.

**Figure 3:**
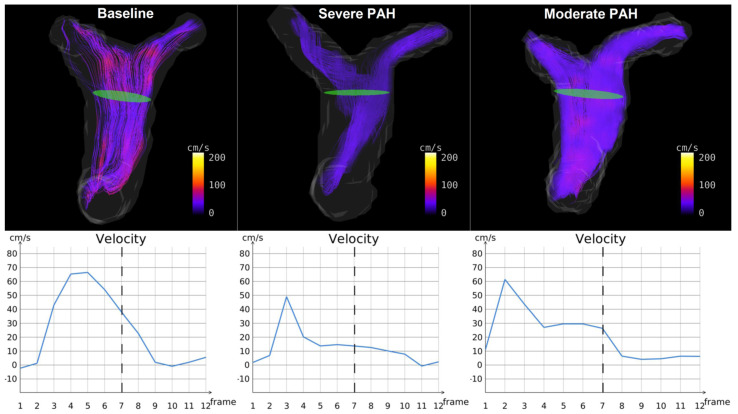
Representative streamline reconstructions of baseline, moderate, and severe pulmonary arterial hypertension in an animal model (top row) with corresponding velocity changes over time, averaged across the green cross-section in the distal MPA (bottom row). The vortical flow pattern formation and its effect on the corresponding velocity-time curve can be observed in this example in the green cross-section area.

**Figure 4:**
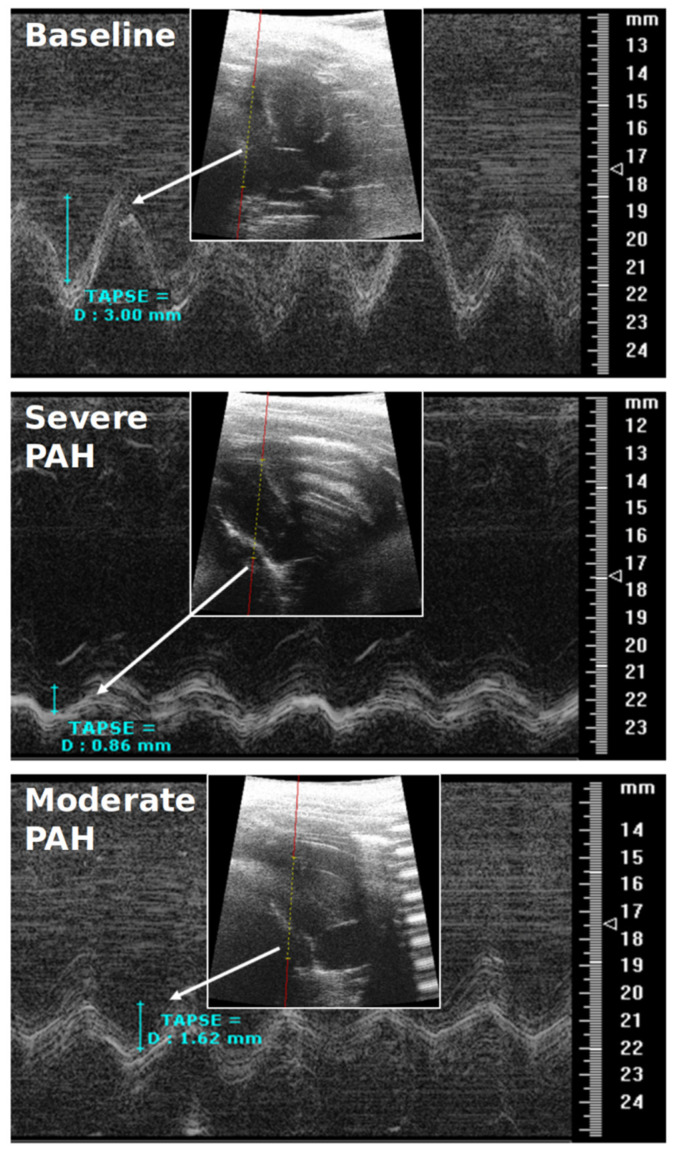
Representative transthoracic echocardiography (TTE) images showing the tricuspid annular plane systolic excursion (TAPSE) in baseline, moderate, and severe pulmonary arterial hypertension.

**Figure 5:**
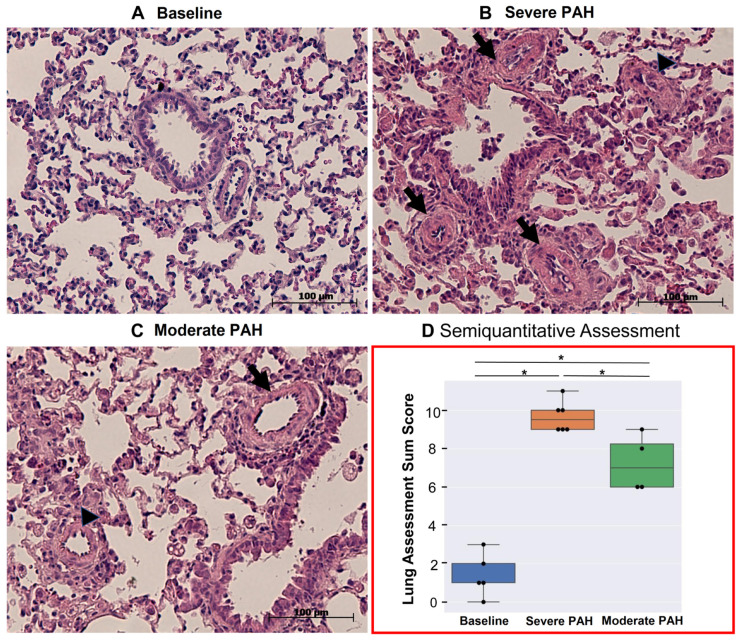
Representative images of histological changes in the lung tissue of baseline, moderate, and severe pulmonary arterial hypertension (PAH) with a focus on media hypertrophy of peribronchial (black arrows) and small arteries (black arrowheads) at a 20× magnification, as well as a graphical comparison between histological sum-score changes between all three experimental groups (* indicates statistical significance, with *p* < 0.05).

- **CMR-Derived Hemodynamics:** The time-averaged mean-velocities (V_mean_) of the large and small pulmonary arteries were investigated in all the experimental groups. In addition, the right and left pulmonary arteriovenous pressure gradients (right: RPSA-RPV_∆P-mean_, left: LPSA-LPV_∆P-mean_) were calculated (Table 1). The V_mean_-related indices in the large pulmonary arteries were only capable of distinguishing between severe PAH and baseline (*p* < 0.05), while the V_mean_-related indices in the small pulmonary arteries were capable of differentiating not only between severe PAH and baseline, but also between the moderate and severe stages of the disease (*p* < 0.05). Notably, RPSA-RPV_∆P-mean_ and LPSA-LPV_∆P-mean_ could differentiate between baseline and severe PAH as well as the moderate and severe disease groups (*p* < 0.05), but with stronger statistical properties than V_mean_, suggesting their suitability for the non-invasive diagnosis and follow-up of PAH.
  The qualitative assessments of the velocity-time curves in the pulmonary arteries indicated that the early and peak systolic velocities did not generally change in PAH. However, the late systolic velocities appeared more attenuated and flattened; i.e., velocity notches occurred (Figure 2, Figure 3 and Supplementary Material Video S1), which affected the V_mean_ values. This phenomenon was more dominant in the small pulmonary arteries compared to the large ones.
- **RVEF & TAPSE:** Due to the pitfalls and difficult procedure of RHC in rats, RVEF and TAPSE were investigated by CMR and TTE to prove all the animals were influenced by PAH. RVEF could reliably differentiate all the experimental groups from each other (*p* < 0.05), i.e., it was largely reduced in severe PAH and preserved in the moderate stage (Table 2). TAPSE could also differentiate well between baseline and severe PAH as well as between the severe and moderate disease groups (*p* < 0.05) (Table 2) (Figure 4). The RVEF and TAPSE results proved the effectiveness of the experimental design.
- **Histological Assessment of Lung Tissue:** In general, the animals with severe PAH demonstrated the highest individual scores in all the histological criteria except for emphysema among all the experimental groups. Therefore, severe PAH showed extensive signs of histological damage compared to baseline through significantly higher sum-scores (*p* < 0.05) (Figure 5). In moderate PAH, the sum-score was significantly lower compared to the severe group but still higher than the baseline (*p* < 0.05), reflecting a partial improvement in histological damage. With respect to the individual indices, moderate PAH showed a significant decrease for atelectasis and media hypertrophy in both the peribronchial and small pulmonary arteries compared to the animals at the severe stage of the disease (*p* < 0.05). However, the media hypertrophy of both arteries was still increased in severe and moderate PAH compared to baseline (*p* < 0.05) (Figure 5).
- **Correlation analysis between 4D-flow parameters and histopathology:** In general, the correlation analysis between the V_mean_-related indices and the histological results showed moderate-to-strong correlation coefficient values, while the correlation analysis between the right/left pulmonary arteriovenous pressure gradients and histological results indicated a strong-to-very-strong associativity (Table 4).

## 4. Discussion and Conclusions

The limitations of RHC in the diagnosis of PAH in combination with its invasiveness prompted us to investigate the potential role of new hemodynamic indices derived from 4D-flow to diagnose PAH. In current research, CMR is used to assess the response of PH to surgical or medical treatments primarily through conventional indices (e.g., right ventricular functions, right ventricular mass, main pulmonary velocities, etc.) [16]. In addition, the hemodynamic changes in the large pulmonary arteries under PH have been extensively assessed in previous studies and are well summarized by Reiter U. et al. [19]. In PAH, we observed the same findings as in the previous clinical studies [21,22,26,27,28,29,30,31], indicating that we reached the same level of accuracy. In a recent study by Cerne et al. [32], the V_mean_ of the main, right and left pulmonary arteries showed considerably lower values in PAH in comparison to the normal population. However, in the current study, we hypothesized that the small pulmonary arteries were more suitable regions than large vessels for the evaluation of hemodynamic changes due to their proximity to the site of media hypertrophy. In contrast to [32], we chose histology as the reference standard instead of RHC to directly evaluate the lung tissue changes in PAH, since hemodynamics alter secondary to histological changes. In addition, we minimized the time gaps between all the diagnostic procedures and kept all the study conditions constant throughout the experiment to mitigate any possible confounding factors arising in between. In our results, the V_mean_ of the small pulmonary arteries differentiated between the baseline, moderate, and severe PAH groups more sensitively than the V_mean_ of the large pulmonary arteries, suggesting its superiority as a hemodynamic index in diagnosing PAH.

In addition, the pronounced changes in the V_mean_ of the small pulmonary arteries in severe PAH reflected the hampered blood flow within the pulmonary circulation as a result of vascular resistance, which led to blood flow pattern changes and vortex ring formation in the large pulmonary arteries (Figure 2 and Figure 3, Supplementary Material Video S1). Vortex rings influenced the shape of the velocity–time curves and could adequately explain the formation of the velocity notches during the late systole, which was also described in [19,33]. Figure 3 and Supplementary Material Video S1 highlight an exemplary case, illustrating the effects of vortex ring formation on the velocity–time curves in the distal portion of the main pulmonary artery. The study by Reiter G. et al. [34] also reported a correlation between vortex ring formation and mean pulmonary artery pressure changes in PH, which supports our observations.

In addition to the evaluation of the V_mean_ in the small pulmonary arteries, we introduced the time-averaged pulmonary arteriovenous pressure gradient as a new diagnostic index. We modeled the pulmonary circulation in the lungs as a simple water stopcock system with one inlet and one outlet (i.e., the vascular stiffness of the lung was modeled as the stopcock, the pulmonary artery was the inlet, and the pulmonary vein was the outlet) and used the modified Bernoulli’s equation to roughly measure the associated pressure gradient caused by the stopcock stiffness, which was equivalent to the severity of media hypertrophy in our model of PAH. This study showed that the pulmonary arteriovenous pressure gradient was one of the most sensitive indices reflecting the severity of PAH. The correlation between this novel parameter and the lung assessment sum-score in histology was found to be very strong (more than 90%), which is superior to all the previously introduced imaging biomarkers summarized in [19].

As stated in the Materials and Methods section, the target regions of this study were the small pulmonary arteries. To meet the necessary spatial properties of such small geometries, the resolution and the measurement noise had to be increased; however, the use of the 4D-flow stack-of-stars velocity mapping technique instead of the 4D-flow Cartesian technique (which is the most frequently used sequence in preclinical CMR) could compensate for the loss of velocity-to-noise ratio and could decrease the measurement bias in these small vessels.

The major pitfall of this study was the high standard deviations in different hemodynamic indices observed in moderate PAH. A reasonable explanation could be that the response to medication was subjective and could vary among individual animals, which might have led to large standard deviations in the results of this group. One suitable remedy for this problem could be an increase in the sample size to allow more accurate statistics. In addition, we recommend a more comprehensive longitudinal study with multiple CMR scans on individual animals at multiple time points during the disease progression, instead of one single time point. Despite all these constraints, we are convinced that the results of the time-averaged pulmonary arteriovenous pressure gradient and the velocity values in the small pulmonary arteries can serve as potentially sensitive biomarkers in the diagnosis and follow-up of PAH.

## Figures and Tables

**Table 1:** Results for CMR-derived hemodynamic parameters in the three experimental groups.

MRI-Derived Index	Abb.	Unit	Sig. (*p* < 0.05)	Group (Mean ± Standard Deviation)		
				Baseline	Severe	Moderate
Main pulmonary artery averaged velocity	MPA_V-mean_	cm·s^−1^	1	30.43 ± 7.90	12.18 ± 4.32	22.28 ± 11.41
Right pulmonary artery averaged velocity	RPA_V-mean_	cm·s^−1^	1	23.15 ± 4.97	13.78 ± 7.24	19.15 ± 10.78
Left pulmonary artery averaged velocity	LPA_V-mean_	cm·s^−1^	1	22.65 ± 6.94	12.77 ± 2.23	20.44 ± 8.11
Right pulmonary small artery averaged velocity	RPSA_V-mean_	cm·s^−1^	1	18.40 ± 3.72	7.43 ± 3.40	14.36 ± 7.62
Left pulmonary small artery averaged velocity	LPSA_V-mean_	cm·s^−1^	1, 3	22.57 ± 4.66	7.47 ± 4.07	16.16 ± 8.50
Right pulmonary arteriovenous averaged pressure	RPSA-RPV_∆P-mean_	mmHg	1, 3	0.12 ± 0.06	0.018 ± 0.02	0.093 ± 0.095
Left pulmonary arteriovenous averaged pressure gradient	LPSA-LPV_∆P-mean_	mmHg	1, 3	0.19 ± 0.09	0.02 ± 0.02	0.11 ± 0.14

1, Two independent Mann–Whitney U tests between baseline and severe groups were significant (*p* < 0.05). 2, Two independent Mann–Whitney U tests between baseline and moderate groups was significant (*p* < 0.05). 3, Two independent Mann–Whitney U tests between severe and moderate groups were significant (*p* < 0.05).

**Table 2:** Results for different right heart functional indices derived from CMR and TTE in the three experimental groups.

Functional Index	Abb.	Unit	Sig. (*p* < 0.05)	Group (Mean ± Standard Deviation)		
				Baseline	Severe	Moderate
**CMR:** Right ventricle ejection fraction	RVEF	%	1, 2, 3	56.27 ± 3.67	17.97 ± 2.31	38.75 ± 10.42
**TTE:** Tricuspid annular plane systolic excursion	TAPSE	mm	1, 2	2.73 ± 0.64	1.03 ±0.17	1.27 ± 0.45

1, Two independent Mann–Whitney U tests between baseline and severe groups were significant (*p* < 0.05). 2, Two independent Mann–Whitney U tests between baseline and moderate groups were significant (*p* < 0.05). 3, Two independent Mann–Whitney U tests between severe and moderate groups were significant (*p* < 0.05).

**Table 3:** Results for pulmonary histological indices in the three experimental groups.

Pulmonary Histopathology Index	Abb.	Sig. (*p* < 0.05)	Group (Mean ± Standard Deviation)		
			Baseline	Severe	Moderate
Atelectasis area	AA	1, 3	0.80 ± 0.44	1.67 ± 0.51	1.00 ± 0.00
Emphysema area	EA	2	0.20 ± 0.447	0.67 ± 0.51	1.25 ± 0.50
Peribronchial artery media hypertrophy	PAMH	1, 2, 3	0.40 ± 0.54	2.83 ± 0.408	2.00 ± 0.00
Peribronchial artery perivascular cellular edema	PAPCE	1	0.00 ± 0.00	1.67 ± 0.816	1.00 ± 1.15
Small artery media hypertrophy	SAMH	1, 2, 3	0.00 ± 0.00	2.83 ± 0.40	2.00 ± 0.00
Lung assessment sum score	LASS	1, 2, 3	1.40 ± 1.14	9.67 ± 0.81	7.25 ± 1.50

1, Two independent Mann–Whitney U tests between baseline and severe groups were significant (*p* < 0.05). 2, Two independent Mann–Whitney U tests between baseline and moderate groups were significant (*p* < 0.05). 3, Two independent Mann–Whitney U tests between severe and moderate groups were significant (*p* < 0.05).

**Table 4:** Results of the inter-parameter correlation analysis.

MRI Index	Corr. Index	AA	EA	PAMH	PAPCE	SAMH	LASS
MPA_V-mean_		−0.537	−0.421	−0.754	−0.866	−0.708	−0.829
RPA_V-mean_		−0.342	−0.383	−0.495	−0.619	−0.550	−0.613
LPA_V-mean_		−0.537	−0.163	−0.723	−0.743	−0.682	−0.817
RPSA_V-mean_		−0.220	−0.529	−0.761	−0.725	−0.816	−0.819
LPSA_V-mean_		−0.342	−0.565	−0.827	−0.785	−0.892	−0.925
RPSA-RPV_∆P-mean_		−0.416	−0.566	−0.792	−0.662	−0.854	−0.868
LPSA-LPV_∆P-mean_		−0.390	−0.526	−0.851	−0.742	−0.917	−0.915

Red: Very strong correlation (r = 0.90 to 1.0); Yellow: Strong correlation (r = 0.70 to 0.90); Green: Moderate correlation (r = 0.50 to 0.70); Blue: weak and negligible correlation (r < 0.50). (MPA_V-mean_: Main pulmonary artery averaged velocity; RPA_V-mean_: Right pulmonary artery averaged velocity; LPA_V-mean_: Left pulmonary artery averaged velocity; RPSA_V-mean_: Right pulmonary small artery averaged velocity; LPSA_V-mean_: Left pulmonary small artery averaged velocity; RPSA-RPV_∆P-mean_: Right pulmonary arteriovenous averaged pressure gradient; LPSA-LPV_∆P-mean_: Left pulmonary arteriovenous averaged pressure gradient).

## Data Availability

The corresponding data related to this study are available upon request.

## Supplementary Materials

The following supporting information can be downloaded at: https://www.mdpi.com/article/10.3390/diagnostics12010058/s1. Video S1: Qualitative assessment of the velocity-time curves in the main pulmonary artery. Due to high vascular resistance in severe pulmonary arterial hypertension (PAH in diseased animals), vortex rings are generated in the large pulmonary arteries producing velocity notches in the corresponding velocity-time curves. This effect was not noticed in either the baseline (normal) or in the moderate PAH (treated) animals.
